# Limitations of Remotely Sensed Aerosol as a Spatial Proxy for Fine Particulate Matter

**DOI:** 10.1289/ehp.0800360

**Published:** 2009-02-21

**Authors:** Christopher J. Paciorek, Yang Liu

**Affiliations:** 1 Department of Biostatistics, Harvard School of Public Health, Boston, Massachusetts, USA; 2 Department of Environmental and Occupational Health, Emory University Rollins School of Public Health, Atlanta, Georgia, USA

**Keywords:** aerosol optical depth, air pollution, geographic information system, predictive modeling, remote sensing, satellite, spatial smoothing, spatiotemporal modeling

## Abstract

**Background:**

Recent research highlights the promise of remotely sensed aerosol optical depth (AOD) as a proxy for ground-level particulate matter with aerodynamic diameter ≤ 2.5 μm (PM_2.5_). Particular interest lies in estimating spatial heterogeneity using AOD, with important application to estimating pollution exposure for public health purposes. Given the correlations reported between AOD and PM_2.5_, it is tempting to interpret the spatial patterns in AOD as reflecting patterns in PM_2.5_.

**Objectives:**

We evaluated the degree to which AOD can help predict long-term average PM_2.5_ concentrations for use in chronic health studies.

**Methods:**

We calculated correlations of AOD and PM_2.5_ at various temporal aggregations in the eastern United States in 2004 and used statistical models to assess the relationship between AOD and PM_2.5_ and the potential for improving predictions of PM_2.5_ in a subregion, the mid-Atlantic.

**Results:**

We found only limited spatial associations of AOD from three satellite retrievals with daily and yearly PM_2.5_. The statistical modeling shows that monthly average AOD poorly reflects spatial patterns in PM_2.5_ because of systematic, spatially correlated discrepancies between AOD and PM_2.5_. Furthermore, when we included AOD as a predictor of monthly PM_2.5_ in a statistical prediction model, AOD provided little additional information in a model that already accounts for land use, emission sources, meteorology, and regional variability.

**Conclusions:**

These results suggest caution in using spatial variation in currently available AOD to stand in for spatial variation in ground-level PM_2.5_ in epidemiologic analyses and indicate that when PM_2.5_ monitoring is available, careful statistical modeling outperforms the use of AOD.

Epidemiologic studies provide evidence that chronic exposure to particulate matter (PM) is related to increased mortality and morbidity (Dockery et al. 1993; Miller et al. 2007; Pope et al. 2002). Studies of the chronic health effects of PM rely on spatial heterogeneity in PM concentrations to identify the effects. Spatial statistical modeling combined with land use regression can improve estimation of concentrations at fine scales by using land use and meteorologic information (Paciorek et al. 2009; Yanosky et al. 2008), but efforts still suffer from the spatial sparsity of the monitoring network.

Remote sensing holds promise for adding spatial information for exposure estimation, particularly in suburban and rural areas far from monitors (e.g., Figure 1). Satellite-derived aerosol optical depth (AOD) is correlated with ground-level PM with aerodynamic diameter ≤ 2.5 μm (PM_2.5_) (Engel-Cox et al. 2004; Koelemeijer et al. 2006; Liu et al. 2005, 2007; Paciorek et al. 2008; Pelletier et al. 2007; Wang and Christopher 2003). These correlations occur despite the vertical mismatch between total column aerosol, as measured by AOD, and ground-level PM_2.5_ the level of interest for health studies, and the temporal mismatch between 24-hr average PM_2.5_ and daytime (often single snapshot) AOD. These results and success in using AOD to document pollution episodes (Al-Saadi et al. 2005; Wang and Christopher 2003) have led to excitement about using AOD as a proxy, standing in for PM_2.5_, or in combination with ground measurements to better predict PM_2.5_. Our attention focuses on improving empirical prediction, rather than physical explanation, of the spatial patterns of PM_2.5_.

Most studies of the AOD–PM_2.5_ association focus on temporal (longitudinal) correlations or do not distinguish spatial (cross-sectional) from temporal correlations, but for chronic exposure, estimating spatial heterogeneity is critical. Correlations of long-term averages using matched daily (or hourly) values (e.g., van Donkelaar et al. 2006) do not take into account the large number of missing retrievals, because of orbit patterns, cloud cover, and surface reflectivity, that may seriously compromise the association between available AOD and long-term average PM_2.5_ concentrations. Finally, but critically, simple correlations do not tell us whether AOD improves predictions within a statistical model that already uses information on meteorology, land use, and regional variation, and we are not aware of any such analysis of the use of AOD for exposure estimation.

Here we report both raw empirical results and statistical modeling of the relationship between AOD and PM_2.5_ and the ability of AOD retrievals to improve predictions of ground-level PM_2.5_ in the eastern United States, focusing on the mid-Atlantic region. We take a public health perspective, in which good estimates of PM_2.5_ concentrations are needed over an entire specified spatial region and time period as an input for epidemiologic analysis. We first show positive, but moderate and variable, correlations at various temporal scales. Correlations do not improve when looking at longer-term averages over all the days in a period of time. We introduce a statistical model that treats AOD as proxy data for PM_2.5_, estimating a PM_2.5_ prediction surface that reflects both the PM_2.5_ and AOD data. This model shows high sensitivity to assumptions about the structure of the discrepancy between AOD and PM_2.5_. The results suggest there are systematic, spatially correlated differences between AOD and PM_2.5_ and that AOD should be disregarded in predicting PM_2.5_. We confirm this using a simpler model with PM_2.5_ data as the gold standard, regressing PM_2.5_ on AOD and numerous other predictors, showing no gain in predictive power from the use of AOD in an already successful prediction model.

## Materials and Methods

### Data

**Figure 1:**
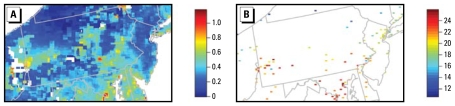
Example of monthly average MODIS AOD (*A*) and ground-level PM_2.5_ from monitors (*B*): July 2004 in our mid-Atlantic study region of the United States.

All analyses are for the year 2004. Associations of AOD and PM_2.5_ are weak in the western United States (Engel-Cox et al. 2004; Liu et al. 2005; Paciorek et al. 2008), so we focus on the eastern United States. Our daily exploratory analyses use data east of 100°W longitude. To limit computations with large remote-sensing data sets, our longer-term analyses, including the statistical modeling, focus on a mid-Atlantic region encompassing Pennsylvania and New Jersey (Figure 1), which contains the major metropolitan areas of New York, New York; Philadelphia, Pennsylvania; Washington, DC; Baltimore, Maryland; and Pittsburgh, Pennsylvania, as well as large rural areas in the north. The heterogeneity in population density and the presence of large point source emissions from power plants and industrial plants in the southwest provide a test region with substantial variability in pollution.

We use AOD retrievals from three satellite instruments: MODIS (moderate resolution imaging spectroradiometer), MISR (multiangle imaging spectroradiometer), and the GOES (Geostationary Operational Environmental Satellite) aerosol/smoke product (GASP). The MODIS and MISR instruments are aboard the Terra satellite platform, whose polar orbit gives full coverage of the globe at regular intervals, starting in March 2000, with retrievals in the eastern United States at a constant daily time point (1030–1045 hours local time). Both MISR (primarily version 15, at 558 nm) and MODIS (collection 5, at 550 nm) provide retrievals of AOD, a dimensionless measure of light extinction over the entire vertical column of air through the atmosphere (also known as aerosol optical thickness). MISR level 2 aerosol data (versions 15 and 17) were downloaded from the U.S. National Aeronautics and Space Administration (NASA) Langley Research Center (LARC) Atmospheric Sciences Data Center (NASA 2006). MODIS aerosol data (collection 5) were downloaded from the MODIS Level 1 and Atmosphere Archive and Distribution System (LAADS) (NASA 2007). AOD generally ranges from 0 to 5, with values > 1 associated with heavy haze. MISR AOD retrievals are at a nominal spatial resolution of 17.6 km with retrievals in the northeast United States every 4–7 days depending on location (Liu et al. 2005). MODIS provides AOD retrievals at a nominal resolution of 10 km with each location covered every 1–2 days (Engel-Cox et al. 2004; Wang and Christopher 2003). AOD cannot be measured below clouds, so cloud filtering algorithms use the infrared portion of the spectrum to detect and omit obscured observations (Engel-Cox et al. 2004). Errors and uncertainties in the filtering can lead to erroneous AOD retrievals, and high surface reflectivity can also prevent retrievals.

GASP AOD (interpolated at 550 nm) is calculated from GOES-12 (East) imager data; the U.S. National Oceanic and Atmospheric Administration (NOAA) provided their most recent version (Knapp et al. 2002; NOAA, personal communication). GASP AOD is at a nominal spatial resolution of 4 km, but retrievals are less precise than MODIS or MISR AOD because of the coarse spectral resolution and fixed viewing geometry of the sensor (Prados et al. 2007). Retrievals are attempted every half-hour during daylight, 1045–2345 hours universal time, but again, cloud cover and high surface reflectivity lead to many missing observations. We use daily average GASP AOD, regardless of the number of retrievals.

We use 24-hr average gravimetric [federal reference method (FRM)] measurements from the U.S. Environmental Protection Agency (EPA) Air Quality System with parameter 88101 (U.S. EPA 2009), omitting a small number of IMPROVE (Interagency Monitoring of Protected Visual Environments) monitors, which tend to be placed where few people live. Although hourly data are better matched in time to the MODIS and MISR snapshots, the number of hourly monitors is limited, and there is no FRM for hourly measurements, plus our interest is in the relationship of PM_2.5_ and AOD at monthly and yearly periods.

In our statistical modeling we use geographic information system (GIS)-based and meteorologic covariates to help explain PM_2.5_ variation, following Yanosky et al. (2008). Covariates that may help predict PM_2.5_ at fine spatial scale include distance to major roads in three road classes (A1: primary roads, typically interstates; A2: primary major, noninterstate roads; and A3: smaller, secondary roads, usually with more than two lanes). We also have point locations of year 2002 primary PM_2.5_ emissions from U.S. EPA’s 2002 National Emissions Inventory (NEI) (U.S. EPA 2006). We calculated other covariates using a GIS at the resolution of the 4-km grid used in our statistical modeling. These include road density in the three road classes, population density, and elevation at the cell centroid. As a measure of nonpoint source emissions in each cell, we assign the density (total emissions divided by county area) of the 2002 NEI area-level primary PM_2.5_ emissions in the county of the cell centroid. We based meteorologic variables on the North American Regional Reanalysis (NARR) (Mesinger et al. 2006) fields, available at 32-km resolution every 3 hr. For each 3-hr value and each grid cell, we computed an inverse distance-weighted average of the NARR values from the four nearest NARR points to the cell centroid. We then averaged values to the month. Our second statistical model uses wind speed and temperature, but we also considered relative humidity (RH), planetary boundary layer (PBL) height, mean sea-level pressure, and precipitation.

We also used a calibrated AOD variable (Paciorek et al. 2008), which accounts for systematic effects of PBL, RH, season, and time-invariant regional variation that modify and obscure the relationship between daily PM_2.5_ and daily AOD. The calibration is done by regressing daily AOD values from 2004 across the eastern United States on daily PM_2.5_ and the variables just mentioned, matched in space and time. By including time-invariant regional variation, we cause the long-term average AOD and long-term average PM_2.5_ to more closely match at large spatial scales, necessarily increasing correlations of PM_2.5_ and AOD. Our hope in including this spatial term is to adjust for large-scale differences between AOD and PM_2.5_, allowing us to exploit common patterns of AOD and PM_2.5_ at smaller spatial scales, to the extent that they exist.

### Exploratory analyses

Our goal in the exploratory analyses was to understand the association between AOD and PM_2.5_ at different temporal aggregations to assess the potential of AOD to help predict chronic PM_2.5_ exposure. We started by considering associations at the daily level when AOD and PM_2.5_ are matched such that both are available for a given day and location, mirroring analyses in the published literature. We matched available PM_2.5_ 24-hr averages with AOD retrievals from the nearest pixel for each of the three satellite instruments, omitting a small number of monitors for which the nearest pixel centroid is closer to another monitor. Our interest is in fine resolution estimation of PM_2.5_, so unlike other analyses we used individual pixels instead of aggregating AOD across adjoining pixels.

When considering prediction of long-term average PM_2.5_ for chronic epidemiologic analyses, missing AOD retrievals cause one to rely on a subset of days (determined by weather conditions that also affect PM_2.5_ levels, so AOD patterns represent only cloud-free conditions) with AOD retrievals to estimate monthly or yearly pollution. Over land, MODIS, MISR, and GOES retrievals are available, on average over the entire mid-Atlantic region, 16%, 4%, and 38%, respectively, of the days in 2004. Also, for MODIS and MISR, the occurrence of AOD snapshots at the same time every day may not well match daily average pollution. To assess the long-term spatial relationship of AOD and PM_2.5_, we considered associations of yearly PM_2.5_ and AOD, relating average AOD from available retrievals to average PM_2.5_ based on all available PM_2.5_ monitoring, not just PM_2.5_ data matched by day to AOD retrievals. These associations eliminate temporal correlations within a site that can obscure the spatial association. However, simple yearly averaging does not account for the differential frequency of successful AOD retrievals over the seasons in the year (which overweights summer AOD values) or allow us to consider monthly associations, so we report results at the monthly level in the Supplementary Material (http://www.ehponline.org/members/2009/0800360/suppl.pdf).

### Statistical modeling

The exploratory analyses do not account for complications such as differing numbers of PM_2.5_ observations and AOD retrievals by location and very fine-scale heterogeneity in PM_2.5_. Most important, correlations of AOD and PM_2.5_ may reflect variability in PM_2.5_ that could be predicted by other sources of information, such as land use or meteorology or estimation of large-scale regional variation through spatial smoothing of monitored values, so they may overstate the usefulness of AOD as a predictor in light of other readily available information. To address these issues we turn to formal statistical modeling, analyzing the mid-Atlantic region. Both models are specified in a Bayesian context and are fitted by standard Markov chain Monte Carlo methods. We did not use MISR because of the limited number of retrievals.

### Using AOD as proxy data

Recent statistical efforts have focused on combining multiple sources of information by treating the sources as reflecting a true, unknown spatial process (Fuentes and Raftery 2005; Gelfand and Sahu, in press; McMillan et al. 2009). Accordingly, we fit statistical models for individual months in which PM_2.5_ and AOD observations are considered to be separate data sources that reflect the unknown PM_2.5_ surface for a given month. The first stage of the model contains two likelihood terms representing the probabilistic relationships of the PM_2.5_ and AOD data to the underlying processes and covariates. For PM_2.5_, for an individual month, we specify the likelihood,


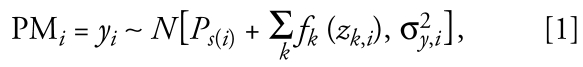


where the core of the model is the unknown true pollution surface that we want to estimate, represented on a 4-km grid as *P_s_*, where *s* indexes grid cells. We represent the monthly averages of available 24-hr concentration measurements in terms of the gridded pollution surface, locating individual observations, *y_i_*, indexed by location *i*, within the appropriate grid cell, *s*(*i*). *f_k_*(*z_k,i_*) are smooth regression functions that reflect the effects of local covariates, *z_k_*, that affect PM_2.5_ at scales below 4 km, a decomposition similar to that of Beelen et al. (2007). In particular, we use distance to the nearest A1 and A2 roads, forcing the effect to be zero beyond 500 m (Zhou and Levy 2007). By modeling the effect of nearby roads (and point emissions), we attempt to account for differences between AOD and PM_2.5_ caused by fine-scale heterogeneity captured by PM_2.5_ monitors but smoothed over in the AOD pixel-level values. Thus, we assess the ability of AOD to capture spatial heterogeneity in PM_2.5_ at small scales (tens of kilometers) but not extremely fine scales (meters to kilometers). σ*_y,i_*^2^ reflects various components of uncorrelated error and accounts for the varying number of daily observations by location. We present below the likelihood term for AOD.

The unknown pollution process on the grid is represented as


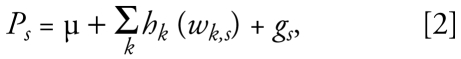


where μ is an overall mean and *h_k_*(*w_k_*_,_*_s_*) are smooth regression functions of grid-scale covariates: the density of A1, A2, and A3 roads, population density, elevation, and nonpoint-source area emissions. *g_s_* is a smooth spatial term representing residual spatial structure unaccounted for by covariates, in particular regional variation. Because we fitted the model individually for each month, we omitted meteorologic covariates, which tend to be spatially smooth and whose influence would be difficult to separate from *g_s_*, causing their influence to be reflected in the estimate of *g_s_*. Also included in the model is a smooth term that accounts for the effect of point emissions within 100 km, where the effect declines with distance and is estimated from the data within the model fitting. This term is used both as a covariate affecting the individual PM_2.5_ observations based on the point location of each monitor (in Equation 1) and as a covariate affecting the gridded process, *P_s_* (based on averaging over a subgrid of 16 points within each cell).

We specify the AOD retrievals in an individual month as reflecting the unknown PM_2.5_ process,


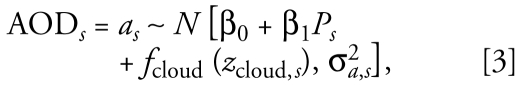


up to additive (β_0_) and multiplicative (β_1_) bias, with a smooth regression function of cloud cover, *f*_cloud_(*z*_cloud,_*_s_*), where *z*_cloud,_*_s_* is the monthly average proportion of cloud-free retrievals in the cell, based on the GOES cloud retrieval algorithm. We included this to help account for bias from retrievals systematically missing because of clouds (Koelemeijer et al. 2006; Paciorek et al. 2008). σ*_a,s_*^2^ reflects various components of uncorrelated error and accounts for the varying number of daily retrievals by location. A complicating factor is that for different satellite orbits on different days, the MODIS pixels shift spatially. Therefore, we consider the overlap of all the pixels in an orbit with the 4-km grid, assigning to each grid cell, *s*, the value of the MODIS pixel in which the cell centroid falls. Taking the retrievals assigned to each cell, we then average to the monthly level for each cell, giving *a_s_*. For GOES the pixel locations are constant over time, so we average to the monthly level and then assign each grid cell the weighted average AOD of the GOES pixels that the cell overlaps, weighted by the area of overlap. Although they are simplistic, we believe these approaches cause minimal distortion in the AOD values used in the modeling, because of the reasonably smooth local variation in daily AOD values from pixel to pixel. In this model, we assume any difference between AOD and PM_2.5_ is spatially uncorrelated noise, which causes estimation of *P_s_* to reflect the spatial structure in both PM_2.5_ and AOD observations.

However, maps of monthly average AOD show strong spatial structure (e.g., Figure 1A) with limited spatially uncorrelated noise (i.e., white noise) apparent. This spatial structure may be caused in part by systematic, spatially correlated differences between AOD and PM_2.5_, rather than reflecting spatial structure in ground-level PM_2.5_. Factors likely to contribute to such differences, which would operate even if AOD were measured perfectly, include spatial structure in pollution aloft above the boundary layer and daily spatial patterns of missing retrievals from clouds with aggregate effect at the monthly level. Of course, AOD is not measured perfectly (Knapp et al. 2002; Remer et al. 2005), as reflected in moderate correlations between monthly average MODIS and GOES AOD and induced in part by spatial variability in surface reflectivity and PM_2.5_ composition. The summed effect of all these differences, which we refer to as systematic discrepancy, could be substantial, and this, rather than pixel-scale white noise, may be the dominant factor explaining low correlations with PM_2.5_ seen in our exploratory analyses. Models that treat AOD as a proxy for PM_2.5_ without accounting for potential systematic discrepancies may predict spatial patterns of PM_2.5_ that do not match reality. We assessed sensitivity to assumptions about systematic discrepancies by including an additive spatial bias term, ϕ*_s_*, represented at the grid scale, replacing the constant bias, β_0_, in Equation 3. Models that include such a term allow for the possibility that AOD retrievals are telling us about spatial processes specific to the retrievals that do not reflect spatial patterns in ground-level PM_2.5_. We estimated ϕ*_s_* using a penalized thin-plate spline approach that penalizes complex spatial surfaces, thereby favoring simple surfaces if the data can be sufficiently well explained by a smooth surface (Ruppert et al. 2003). We also used such an approach for the other smooth terms in the model, fitted naturally within the Bayesian context with the level of smoothing determined by the data. For computational reasons and because the key results are best visualized in model fits of individual months, we fitted the model separately for each of the 12 months.

The advantage of this modeling approach is that it naturally treats AOD retrievals as data and allows for missing retrievals. By considering different assumptions about spatial bias, we can assess the concordance of spatial patterns between AOD and PM_2.5_ and investigate the assumption that the spatial pattern in AOD represents signal that is informative about PM_2.5_.

### Using AOD as a predictor of PM_2.5_

We also consider a model in which AOD is used as a predictor on the right-hand side of a regression-style model, treating the PM_2.5_ data as the gold standard. This has the benefit of directly calibrating PM_2.5_ to AOD and, if there is little empirical association, discounting AOD as a predictor of PM_2.5_.

In this model, we modeled PM_2.5_ observations as in Equation 1,


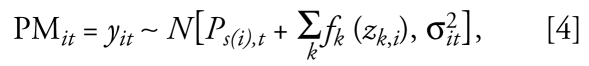


whereas the unknown smooth pollution process, *P_s_*_,_*_t_*, is similar to Equation 2 but includes AOD, *A_s_*_,_*_t_*, as a predictor:


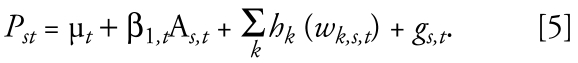


This model is fit simultaneously to all 12 months, indexed by the *t* subscript. For simplicity, we assume that *g_s_*_,_*_t_*, the residual spatial structure, is not correlated over time, which eases computations. Previous work suggests month-to-month correlation is limited and that including correlation would do little to improve predictions (Paciorek et al. 2009), so the assumption should not affect our ability to assess whether AOD can improve PM_2.5_ predictions. We allow β_1,_*_t_* to vary in an unstructured way with time in case the relationship of AOD and PM_2.5_ varies by season (Paciorek et al. 2008). Based on some limited variable selection, the covariates *w_k_*_,_*_s_*_,_*_t_* (some of which do not vary with time) are population density, elevation, area emissions, point emissions, density of A3 roads, wind speed, and temperature. We also include monthly average cloudiness to help account for bias from missing AOD retrievals.

For this approach, a downside is that we require AOD values at all locations. We used the Markov random field approximation to a thin-plate spline described in the Supplementary Material (http://www.ehponline.org/members/2009/0800360/suppl.pdf) to smooth the observed AOD retrievals and make predictions, *A_s_*_,_*_t_*, at unobserved locations. Preprocessing of pixel-level AOD values to align with the 4-km grid is as described previously.

## Results

### Exploratory analyses

Correlations between daily PM_2.5_ and AOD (matched by day and location) that reflect both temporal and spatial associations are higher than correlations for individual days, taken across spatial locations (Table 1), which reflect only spatial associations. The spatiotemporal associations roughly match those seen in the literature that have been used as evidence of the potential of AOD as a proxy for PM_2.5_ (e.g., Engel-Cox et al. 2004; Liu et al. 2005; Paciorek et al. 2008). Using AOD directly does not account for meteorologic factors and systematic temporal and spatial variability that modify the relationship between AOD and PM_2.5_, so we also considered the calibrated version of AOD, which somewhat improved the correlations (Table 1).

Table 1 shows near-zero correlations of yearly average PM_2.5_ from all available 24-hr values (everyday or every-third-day sampling) with AOD from available retrievals. Note that for monitors reporting only every 3 days, missing PM_2.5_ values contribute to noise in the associations seen here. After calibration, AOD is moderately correlated with PM_2.5_. The calibration includes an overall spatial term, adjusting for any large-scale regional mismatch between AOD and PM_2.5_ that is consistent over the year. This term is responsible for much of the increase in correlation after calibration because it necessarily causes the large-scale patterns of long-term average AOD and PM_2.5_ to more closely match. Our hope is that correcting for such large-scale mismatch allows us to explore whether there is independent information in AOD for predicting smaller-scale patterns of PM_2.5_, a question answered in the statistical modeling. For results at the monthly level, see the Supplementary Material (http://www.ehponline.org/members/2009/0800360/suppl.pdf).

### Using AOD as proxy data: sensitivity to systematic discrepancies

**Figure 2:**
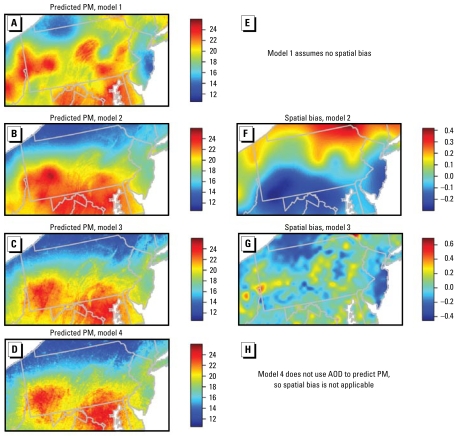
Sensitivity of predicted PM to the characterization of spatial bias. The left column shows PM predictions for models in which AOD and PM observations are treated as data reflecting a common unknown PM process, using calibrated MODIS AOD for July 2004. (*A*) Model 1: excluding the spatial bias term, ϕ*_s_*, thereby treating AOD as a simple proxy for PM with simple additive and multiplicative bias. (*B*) Model 2: ϕ*_s_* constrained to be a somewhat smooth process with a maximum of 55 degrees of freedom (df) (a penalized spline with 55 knots). (*C*) Model 3: ϕ*_s_* relatively unconstrained with a maximum of 755 df. (*D*) Model 4: AOD not used. The right column shows the corresponding estimated ϕ*_s_* surfaces, except that for model 1, ϕ*_s_* is not included in the model (*E*); for model 4 AOD is not used, so ϕ*_s_* is not involved in the model (*H*). (*F* and *G*) Spatial discrepancy for models 2 and 3, respectively.

For July 2004 for MODIS AOD, Figure 2 shows model-based predictions of PM_2.5_ and estimates of ϕ*_s_* based on Equations 1–3, allowing different amounts of complexity in ϕ*_s_*. When the model omits the spatial bias term (Figure 2A,E), representing AOD as reflecting PM_2.5_ up to simple additive and multiplicative bias, predictions of PM_2.5_ strongly track AOD spatial patterns (i.e., Figure 1A). As we introduce spatial bias (Figure 2B,F) and allow more flexibility in the spatial bias term (Figure 2C,G), predictions increasingly track the PM_2.5_ observations (i.e., Figure 1B) and results from a model fitted without AOD (Figure 2D,H). The fit of the penalized spline model does not stabilize on a smooth bias surface. When we force the bias term to be smooth, the model cannot adequately represent the AOD data based on the PM_2.5_ surface, the smooth bias, and white noise error. This suggests there is little common spatial pattern to PM_2.5_ and AOD observations and that true PM_2.5_ is best modeled solely based on ground-level PM_2.5_ data with AOD variability modeled separately. This is demonstrated in Figure 2C and G, where the model essentially disregards AOD in predicting PM_2.5_ and attributes most of the variability in AOD to ϕ*_s_*. Results for the other 11 months and using GOES AOD or raw AOD give similar conclusions. In summary, systematic discrepancies are considerable and critical to include, and predictions are very sensitive to assumptions about the discrepancy term. If the spatial discrepancy were estimated to be a relatively smooth process, able to be resolved from having PM_2.5_ and AOD data in the same region, the modeling approach provides a means to improve PM_2.5_ prediction by combining the data sources while accounting for the discrepancy. However, these results suggest the discrepancy process is not smooth and cannot be adequately estimated without denser PM_2.5_ data, which are not available and would largely obviate the need for AOD as a proxy.

### Using AOD as a predictor: effects on predictive ability

With AOD as a predictor, predictive ability at both the monthly and yearly resolutions does not improve when either calibrated MODIS or GOES AOD is added to the model (Equations 4 and 5) already containing the other predictors (Table 2). If we exclude the other predictors (except the GOES cloud term for consistency in comparing the AOD and no-AOD models) and account for spatial variability solely based on spatial smoothing of the observations within the model framework, addition of AOD still shows essentially no improvement in predictions (Table 2). Results are similar when avoiding locations that are most likely affected by very local sources (Table 2). Sensitivity analyses indicate that there was similarly limited effect of AOD on predictive power when using raw AOD instead, when restricting to monitors in areas with sparse monitoring, or when restricting to everyday monitors (which avoids the extra noise caused by missing monitor values) (data not shown). The higher predictability of monthly compared with yearly PM_2.5_ in Table 2 occurs because of the importance of temporal variation, which is easy to estimate based on the monitoring. The results of including AOD are consistent with the estimates of β_1,_*_t_*, which are small in magnitude, with wide uncertainty intervals that cover zero. Correlations of predictions with and without AOD are > 0.999, indicating that there would be negligible impact in an epidemiologic analysis.

## Discussion

We urge caution in assuming that currently available remotely sensed AOD can help improve exposure estimation for PM_2.5_ and particular caution in using AOD to estimate spatial heterogeneity where there is little ground-level PM_2.5_ data for ground truthing, based on the lack of strong spatial correlation between available AOD retrievals and long-term average PM_2.5_. In a setting in which reasonably dense PM_2.5_ data are available, our statistical modeling results indicate little or no improvement in prediction of long-term average PM_2.5_ when adding AOD. To the extent that raw correlations of AOD and PM_2.5_ reflect the ability of AOD to capture some of the pattern in PM_2.5_, our results suggest that these can be better estimated by simple spatial smoothing of the available PM_2.5_ data and regression on other predictors, rendering the AOD information extraneous. Koelemeijer et al. (2006) found much stronger correlations of yearly average MODIS AOD and PM_10_ in Europe; this may be related to their focus on rural background sites, their larger spatial domain, and the greater variability in their PM_2.5_ concentrations.

Remote sensing is of particular interest in developing countries with little monitoring (e.g., Kumar et al. 2008), but our results suggest that spatial patterns seen in AOD may poorly reflect spatial patterns in ground-level PM_2.5_. Without evidence of strong correlations over space, as opposed to purely temporal correlations, use of AOD to determine spatial heterogeneity in PM_2.5_ may be misleading. Given our focus on a region of moderate size, it is possible AOD would be more helpful for larger regions, although daily spatial correlations over the eastern United States are relatively weak (Table 1), and previous work shows at best moderate long-term correlations over the United States (Rush et al. 2004). AOD might be helpful for estimating temporal heterogeneity, but missing AOD is a major problem.

One might ask whether AOD is useful under specific conditions or in specific locations, such as for pollution episodes (e.g., Wu et al. 2006). It is not clear how important such episodes are for long-term average PM_2.5_ prediction or how to include such information only under the circumstances in which it is predictive of PM_2.5_. To the extent to which AOD is useful in some but not all circumstances, the practical challenge is the need of epidemiologists for exposure estimates without gaps in space or time, often over large domains and long periods of time.

Systematic discrepancies such as those in the satellite AOD proxy for PM_2.5_ can easily be misleading because the spatial structure seen in the proxy leads one to think that the patterns reflect real patterns in the process of interest. In this setting, the evidence suggests that much of this structure does not represent true structure in PM_2.5_. Such systematic discrepancies arise in other contexts (Campbell 1996, p. 378; Robinson 2004, pp. 91–92). It seems likely that deterministic model output used to estimate atmospheric processes, including pollution, such as the widely used Community Multiscale Air Quality model, contain systematic errors that induce correlated errors in model output, because of either errors in inputs or aspects of the system under study that are not captured by the model.

Some avenues for potential improvement in using remote-sensing information to predict PM_2.5_ hold promise. First, Liu et al. (2005) and van Donkelaar et al. (2006) report improvements in relationships of AOD and PM_2.5_ when adjusting for the vertical mismatch based on vertical profile information from an atmospheric chemistry model. However, even after such adjustment, AOD missingness continues to be a problem, and this strategy requires expensive and time-consuming long-term model runs. MISR retrieval information, because it provides reflectivity at multiple angles, and ground light detection and ranging (LIDAR) (Engel-Cox et al. 2006) might also prove helpful in distinguishing ground-level aerosol from aerosol aloft, but spatial coverage is limited. Second, AOD retrieval algorithms aim to accurately estimate AOD, with comparisons with ground-based observations of AOD from the aerosol robotic network (AERONET). Instead, a tailored approach that modifies AOD retrieval algorithms to directly derive a proxy for ground-level PM_2.5_ may improve upon the current algorithms. Improved characterization of spatial patterns in surface reflectivity and particle composition may be a critical avenue for retrieval algorithm improvement, potentially via statistical approaches that are informed by ground-level PM_2.5_ data. Additional work on improving cloud screening algorithms may also be fruitful if it decreases missingness by omitting fewer retrievals not contaminated by clouds or omits retrievals currently suffering from contamination. It should be noted that several significant improvements have been implemented in the latest GASP AOD retrieval since acceptance of this paper (Kondragunta S, personal communication). These changes, including a refined azimuth angle definition, improved surface reflectance estimation method, and improved standard deviation calculation, may help reduce the noise level in GASP AOD data and therefore enhance its predicting power in our models.

## Figures and Tables

**Table 1:** Correlations of daily AOD with matched 24-hr PM for the eastern United States and yearly average AOD with PM, matched in space, for our mid-Atlantic focal region.

	Raw AOD			Calibrated AOD^a^		
Type of variation	MODIS	MISR	GOES	MODIS	MISR	GOES
Daily values, eastern United States						

Temporal plus spatial variation: overall correlation of daily values across all sites and days	0.60	0.50	0.38	0.64	0.57	0.40
Spatial variation only: average of daily spatial correlations^b^	0.35	0.30	0.23	0.45	0.32	0.29

Yearly averages, mid-Atlantic focal region^c^						

Spatial variation only: correlation of yearly averages	0.09	0.25	−0.07	0.49	0.22	0.53

a Calibrated AOD has been adjusted to account for the effects of PBL, RH, season, and regional variation in modifying the relationship between daily AOD and PM.

b Only days with at least 20 matched sites.

c Yearly averages reflect all available AOD retrievals and all available 24-hr average PM concentrations. Yearly results include only sites with at least 100 daily PM observations and exclude one site with high PM levels outside Pittsburgh that is just downwind of a major industrial facility.

**Table 2:** Cross-validation *R*^2^ (mean squared prediction error) for predictions of yearly and monthly average PM from regression style models with and without calibrated AOD and other predictors.

	Yearly averages^a^,^b^		Monthly averages^a^	
Model	All monitors (*n* = 151)	Population exposure^c^ monitors (*n* = 130)	All monitors (*n* = 1,793)	Population exposure^c^ monitors (*n* = 1,542)
Models including land use, emissions, and meteorologic predictors				

No AOD	0.580 (1.04)	0.570 (0.93)	0.827 (2.71)	0.839 (2.48)
With calibrated MODIS AOD	0.573 (1.06)	0.564 (0.94)	0.825 (2.73)	0.839 (2.50)
With calibrated GOES AOD	0.572 (1.06)	0.563 (0.95)	0.825 (2.73)	0.838 (2.50)

Models without land use, emissions, and meteorologic predictors^d^				

No AOD	0.463 (1.33)	0.456 (1.18)	0.794 (3.22)	0.810 (2.94)
With calibrated MODIS AOD	0.467 (1.32)	0.459 (1.17)	0.794 (3.22)	0.810 (2.94)
With calibrated GOES AOD	0.467 (1.33)	0.458 (1.17)	0.794 (3.22)	0.810 (2.94)

a For a given location, only months for which the location has at least four PM daily values are included. Results exclude one site with high PM values outside Pittsburgh that is just downwind of a major industrial facility.

b Yearly average results include only locations with at least 6 available months of PM data.

c The “population exposure” designation assigned to monitors by U.S. EPA indicates that such monitors are not likely to be affected by large, local sources.

d These models include the GOES cloud term for consistency of comparisons between the AOD and no-AOD models.
